# Air pollution and the risk of second primary lung cancer among lung cancer survivors: the prospective UK Biobank cohort study

**DOI:** 10.1038/s41416-026-03454-6

**Published:** 2026-04-27

**Authors:** Eunji Choi, Sophia Luo, Victoria Y. Ding, Anna Graber-Nadich, Julie T. Wu, Rita Popat, Iona Cheng, Joel W. Neal, Heather A. Wakelee, Summer S. Han

**Affiliations:** 1 Department of Population Health Sciences, Weill Cornell Medicine, New York, NY USA; 2 Quantitative Sciences Units, Stanford University School of Medicine, Stanford, CA USA; 3 Department of Epidemiology and Population Health, Stanford University School of Medicine, Stanford, CA USA; 4 Veterans Affairs Palo Alto Health Care System, Palo Alto, CA USA; 5 Department of Epidemiology and Biostatistics, University of California, San Francisco, CA USA; 6 Division of Oncology, Department of Medicine, Stanford University School of Medicine, Stanford, CA USA; 7 Department of Neurosurgery, Stanford University School of Medicine, Stanford, CA USA

**Keywords:** Cancer epidemiology, Lung cancer, Risk factors

## Abstract

**Background:**

Lung cancer survivors have a high risk of second primary lung cancer (SPLC). While air pollution is associated with the risk of initial primary lung cancer (IPLC), especially in never smokers, its effect on SPLC risk is unknown.

**Methods:**

We identified 2439 IPLC patients from the UK Biobank, followed through 2017, linking baseline addresses to 2005–2007 annual average exposures of particulate matter (PM10) and nitrogen dioxide (NO_2_) from the EU-wide Land Use Regression model. Associations with SPLC risk were assessed using cause-specific Cox models adjusted for co-pollutants, socioeconomic factors, smoking, and tumour characteristics.

**Results:**

Of 2439 IPLC patients, 92 (3.7%) developed SPLC over 6561 person-years. The 10-year cumulative incidence of SPLC was 3.98% (3.11–4.85%). A dose-response relationship was observed between PM10 and SPLC risk, with an adjusted hazard ratio (aHR) of 6.43 (2.38–17.32) in the highest vs. lowest (aHR = 1.69 [0.68–4.19]) quintile; a co-pollutant NO_2_ showed an aHR of 0.96 (0.93–0.99). The PM10 effect was pronounced in never-smoking (aHR = 2.26 [1.22–4.18]) vs. ever-smoking IPLC patients (aHR = 1.42 [1.21–1.68]) on the risk of non-small cell SPLC.

**Interpretation:**

Exposure to PM10 may increase SPLC risk in lung cancer survivors, highlighting the potential value of incorporating environmental factors into SPLC surveillance to identify high-risk individuals.

## Introduction

Lung cancer is the leading cause of cancer death worldwide [1]. With advances in early detection and therapeutics, the number of long-term lung cancer survivors is rapidly increasing [2]. Recent evidence has shown that lung cancer survivors are subsequently at a high risk of developing second primary lung cancer (SPLC) [3–6], which is 4–6 times higher than the risk of developing initial primary lung cancer (IPLC) in the general population [5, 7]. Further, over 80% of the detected SPLC cases are found in asymptomatic individuals [8], with survivors diagnosed with SPLC having a significantly increased risk of mortality compared to those without SPLC (HR = 2.12; *p* < .001) [9]. These findings highlight the need to identify risk factors for SPLC and to develop a strategy to identify high-risk survivors who should be screened and followed closely.

Several recent studies have examined different risk factors associated with SPLC development [7, 8, 10–13]. However, these studies focused on factors relating to tumour characteristics of IPLC or various smoking-related factors, including smoking pack-years, duration, or cigs smoke per day [12]. Although a recent study has shown that the risk of developing SPLC is similarly high among never-smoking lung cancer survivors as those with a smoking history [14], little is known about the factors that increase SPLC risk among never-smoking lung cancer survivors. Moreover, the potential impact of environmental exposures on the risk of SPLC is poorly understood, as the datasets with the requisite data are generally limited. Most existing studies for SPLC were conducted using single institute-based data [8, 11] or cancer registry data [10, 13] that have limited access to comprehensive environmental exposure data with long-term follow-up.

Outdoor air pollution has been identified as a major contributor to the development of IPLC, particularly among never-smokers [15] diagnosed with non-small cell IPLC. Exposure to ambient air pollutants, including particulate matter (PM) and nitrogen dioxide (NO_2_), is associated with IPLC risk [16–24]. A cohort study of never-smokers from Taiwan found that air pollution level changes could affect the incidence of lung adenocarcinoma [25]. These findings may be attributed to long-term exposure to air pollution, causing increased lung cancer risk through oxidative damage via inflammatory injury and the production of reactive oxygen species. However, while the association between air pollutant exposure and IPLC has been studied, whether continued exposure to air pollution potentially impacts the subsequent development of SPLC remains uninvestigated among those already diagnosed with IPLC (i.e., lung cancer survivors).

In this study, we aimed to examine whether exposure to residential ambient air pollution is associated with the risk of SPLC among lung cancer survivors using a thoroughly adjusted co-pollutant model, utilising data from the large, prospective, population-based UK Biobank cohort study. Moreover, we conducted various subgroup analyses to evaluate the robustness of the finding between air pollution and SPLC risk, and to identify potential effect modification of the air pollution impact on SPLC.

## Study design and methods

### Study population

**Fig. 1:**
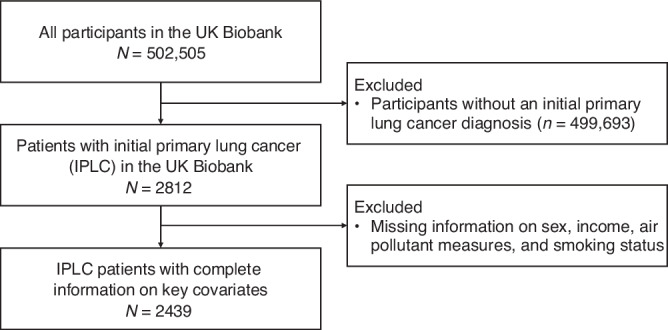
Cohort selection diagram.

The UK Biobank is a prospective cohort study that enrolled around 500,000 individuals aged 40-69 years from 2006 to 2010 to collect deep genetic, physical, and health data. Participants across England, Wales, and Scotland visited assessment centres, where they completed a self-reported questionnaire that asked for comprehensive personal and exposure information, underwent physical and functional measurements, and provided biological samples for assays [26]. Our study cohort consisted of 2439 patients diagnosed with either prevalent (*N* = 275) or incident IPLC (*N* = 2164) in the time between study entry (i.e., 2006–2010) and the last follow-up (i.e., 2017) who had complete information on air pollutant measures, sex, individual-level (household income) and area-level (Townsend Deprivation Index) socioeconomic status, age at IPLC diagnosis, IPLC histology, and smoking status at baseline assessment (Fig. 1). Ethical approval for this study was granted by the Institutional Review Board at Stanford University (IRB #46122).

### Primary outcome

The primary outcome was defined as the time from IPLC diagnosis to SPLC incidence, death (competing risk), or censoring (loss to follow-up or administrative censoring), whichever occurred first. To accurately ascertain SPLC cases and reduce the misclassification between SPLC and lung cancer recurrence, we applied a modified version of the widely-used clinical criteria proposed by Martini and Melamed [7, 9, 27], which classifies a tumour as an SPLC if either (1) the new tumour has a different histology than that of the IPLC, or (2) if the new tumour developed ≥2 years after the time of IPLC diagnosis. Information on IPLC and SPLC diagnosis was obtained via linkage to national cancer registries in England, Wales, and Scotland.

### Ambient air pollution measurement

The annual average estimates of ambient air pollutants, including particulate matter (PM)⩽10 μm in aerodynamic diameter (PM10) and nitrogen dioxide (NO_2_), were calculated by the UK Biobank for each participant based on the address that the participant provided at baseline assessment (2006–2010). Air pollution estimates (i.e., PM10 and NO_2_) for the years 2005–2007 were derived from EU-wide air pollution maps modelled based on a Land Use Regression (LUR) model for Europe.

Additionally, PM⩽2.5 μm in aerodynamic diameter (PM2.5), coarse PM between 2.5 and 10 μm in aerodynamic diameter (PMcoarse), and PM10 and NO_2_ were re-modelled for the year 2010 at each address using a LUR model developed as part of the European Study of Cohorts for Air Pollution Effects (ESCAPE) study (Please refer to **Supplemental Methods** for more information).

For the primary analysis investigating the dose-response relationship, we used quintile estimates of EU-wide air pollution data (PM10) from 2005 to 2007 to ensure a larger number of SPLC cases, taking into account the temporal relationship between exposure (air pollution) and outcome (SPLC development). ESCAPE-based measures (PM10, PM2.5, and PMcoarse) from the year 2010 were applied in sensitivity analyses.

After confirming a dose-response relationship between EU-wide PM10 levels and SPLC risk in the primary analysis, we further examined the effect of linear PM10 on SPLC risk in different subgroups of lung cancer survivors to identify those at higher risk of air pollution-induced SPLC development, since using linear PM10 in these analyses helped avoid convergence issues in small subset analyses.

### Covariates for quantifying the air pollution effect on SPLC risk

Given that air pollutants often coexist, and their concentrations can be correlated, using a co-pollutant model is recommended to isolate the specific effect of the air pollutant of interest. [28] To estimate the impact of PM10 on SPLC risk, we included NO_2_ as a key co-pollutant in the model as a linear term to control for confounding from correlated exposures while maintaining model stability against multicollinearity. Additionally, air pollution concentrations are closely associated with both individual and area-level socioeconomic factors. Thus, we accounted for household income as an individual-level factor and the Townsend Deprivation Index as an area-level socioeconomic factor.

Based on the SPLC literature, we also included smoking status, age at IPLC diagnosis, and IPLC histology as covariates, since these factors are known to be associated with SPLC risk [7, 12]. Information on smoking status was provided by participants at baseline assessment, and they were classified as never, former, or current smokers. Age at IPLC diagnosis and IPLC histology were obtained from national cancer registries linked to each participant’s record in the UK Biobank.

### Statistical analyses

Descriptive statistics, including the number of cases, proportions, and standard deviations, were presented according to outcome status (SPLC, competing-event death, and censored). We estimated the cumulative incidence of SPLC from IPLC diagnosis using the Aalen-Johansen estimator to account for the competing risk of death [29], as most lung cancer survivors are heavy smokers or older individuals and face a high risk of dying from other causes before developing SPLC. The cumulative incidence of SPLC was estimated in entire lung cancer survivors, and by binary PM10 levels, using the World Health Organisation’s high vs. low threshold of 20 µg/m³. Gray’s method was used to assess differences in cumulative incidence across PM10 levels.

To evaluate the effect of air pollutant (PM10) on the risk of SPLC, we applied cause-specific Cox (CSC) regression, with death as the competing event. The CSC function was selected as it can give a valid causal inference in survival data with multiple competing events [30]. Schoenfeld residuals were used to test the proportional hazard assumption, and no violations were detected, indicating that neither air pollution nor other covariates exhibited significant time-dependent effects.

To maintain exposure (air pollution)—outcome (SPLC) temporality when evaluating the association between PM10 and SPLC, we censored the participants who were diagnosed with SPLC prior to when the air pollutant was measured (e.g., excluding participants who were diagnosed with SPLC before 2005 when evaluating PM10 measured in 2005–2007).

### Subgroup analyses

To evaluate the robustness of the finding between air pollution and SPLC risk and to identify potential effect modification of the air pollution impact on SPLC, we evaluated the associations between PM10 and SPLC risk among various sub-populations. First, a restricted analysis was conducted using only those with an incident IPLC after the enrolment in the UK Biobank study (2006–2010) (vs. a combined cohort of prevalent and incident IPLC). Second, we conducted a sub-analysis using only those who lived at the address recorded at baseline for at least 6 months in 2007 and who remained at this location through their IPLC diagnosis and entire follow-up period. Third, given that air pollution effects have been reported to be more significant in never smokers and specific lung cancer histology (e.g., adenocarcinoma), we examined the effect of PM10 based on smoking history (never vs. ever smokers) for overall SPLC and non-small cell SPLC specifically.

### Ethical approval and consent to participate

The UK Biobank study was approved by the Northwest Multi-Centre Research Ethics Committee (reference number 06/MRE09/65), and all participants provided written informed consent at recruitment, including consent for follow-up, via a signature capture device. Data storage and access for this project were strictly controlled in accordance with the data agreements between the researchers and UK Biobank. The study protocol was approved by the institutional review boards of Stanford University (IRB protocol number: 46122). All procedures involving human participants were performed in accordance with the ethical standards of the institutional research committee and with the Declaration of Helsinki and its later amendments or comparable ethical standards. This study is reported in accordance with the STROBE (Strengthening the Reporting of Observational Studies in Epidemiology) guidelines. All methods were performed in accordance with relevant guidelines and regulations. Ethical approval for this study was granted by the Institutional Review Board at Stanford University (IRB #46122).

## Results

The characteristics of the participants at baseline assessment in the UK Biobank cohort are shown in Table 1. Of the 2439 patients diagnosed with IPLC before (*N* = 275) or after enrolment (*N* = 2164) in the UK Biobank Study, with a mean age of IPLC diagnosis of 65.6 years, 36.5% of IPLC patients were current smokers at enrolment.

**Table 1:** UK Biobank IPLC participant characteristics stratified by outcome status.

	Overall (*N* = 2439)		Censored (*N* = 679)		SPLC (*N* = 92)		Competing events			
							Lung Cancer Death (*N* = 1391)		Other-Cause Death (*N* = 277)	
Time, Mean (SD)	2.69	(4.09)	6.22	(5.44)	1.54	(3.50)	1.04	(1.23)	2.71	(4.30)
Sex, *N* (%)										
Men	1269	(52.00)	295	(43.40)	46	(50.00)	765	(55.00)	163	(58.80)
Women	1170	(48.00)	384	(56.60)	46	(50.00)	626	(45.00)	114	(41.20)
Age at IPLC diagnosis, Mean (SD)	65.58	(6.94)	64.13	(8.19)	64.44	(6.71)	66.3	(6.16)	65.84	(6.83)
Histology of IPLC, *N* (%)										
Non-Small Cell Lung Cancer										
Adenocarcinoma	1009	(41.40)	319	(47.00)	39	(42.40)	554	(39.80)	97	(35.00)
Large cell carcinoma	34	(1.40)	12	(1.80)	2	(2.20)	16	(1.20)	4	(1.40)
Squamous	512	(21.00)	164	(24.20)	19	(20.70)	270	(19.40)	59	(21.30)
NSCLC/NOS	226	(9.30)	30	(4.40)	12	(13.00)	168	(12.10)	16	(5.80)
Small Cell Lung Cancer	238	(9.80)	31	(4.60)	7	(7.60)	178	(12.80)	22	(7.90)
Other	420	(17.20)	123	(19.10)	13	(14.10)	205	(14.70)	79	(28.50)
Smoking Status, *N* (%)										
Never	347	(14.20)	132	(19.40)	9	(9.80)	172	(12.40)	34	(12.30)
Former	1205	(49.40)	352	(51.80)	31	(44.60)	671	(48.20)	131	(50.90)
Current	887	(36.49)	195	(28.70)	32	(45.70)	548	(39.40)	102	(36.80)
Smoking pack-years, Mean (SD)	33.49	(26.98)	30.17	(25.94)	30.49	(19.69)	34.57	(27.15)	37.03	(29.72)
TDI, *N* (%)										
1 (<−3.374)	486	(19.90)	158	(23.30)	17	(18.50)	265	(19.10)	46	(16.60)
2 (⩾−3.374 to <−1.840)	489	(20.00)	143	(21.10)	15	(16.30)	276	(19.80)	55	(19.90)
3 (⩾−1.840 to <0.513)	488	(20.00)	123	(18.10)	30	(32.60)	272	(19.60)	63	(22.70)
4 (⩾0.513 to <3.228)	488	(20.00)	121	(17.8)	13	(14.10)	296	(21.30)	58	(20.90)
5 (⩾3.228)	488	(20.00)	134	(19.70)	17	(18.50)	282	(20.30)	55	(19.90)
Household income, *N* (%)										
<18 K	873	(35.80)	218	(32.10)	37	(40.20)	512	(36.80)	106	(38.30)
18K–31K	586	(24.00)	158	(23.30)	21	(22.80)	341	(24.50)	66	(23.80)
⩾31 K	480	(19.70)	158	(23.30)	13	(14.10)	263	(18.90)	46	(16.60)
Unknown	500	(20.50)	145	(21.40)	21	(22.80)	275	(19.80)	59	(21.30)
PM_10_ (2007), Mean (SD)	22.59	(2.60)	22.54	(2.65)	23.4	(2.36)	22.49	(2.530)	22.98	(2.77)
PM_10_ quintile (2007), *N* (%)										
1	488	(20.00)	145	(21.40)	8	(8.70)	293	(21.10)	42	(15.20)
2	491	(20.10)	134	(19.70)	12	(13.00)	290	(20.80)	55	(19.90)
3	487	(20.00)	134	(19.70)	22	(23.90)	277	(19.90)	54	(19.50)
4	485	(19.90)	142	(20.90)	26	(28.30)	256	(18.40)	61	(22.00)
5	488	(20.00)	124	(18.30)	24	(26.10)	275	(19.80)	65	(23.50)
NO_2_ (2007), Mean (SD)	32.07	(10.47)	31.73	(10.88)	32.68	(10.87)	31.85	(10.05)	33.83	(11.25)
PM_2.5_ (2010), Mean (SD)	10.2	(1.08)	10.13	(1.09)	10.18	(1.05)	10.21	(1.07)	10.32	(1.14)
PM_coarse_ (2010), Mean (SD)	6.47	(0.91)	6.5	(0.96)	6.6	(0.98)	6.46	(0.90)	6.42	(0.80)
PM_10_ (2010), Mean (SD)	16.4	(1.86)	16.43	(1.93)	16.63	(1.80)	16.37	(1.84)	16.33	(1.76)
NO_2_ (2010), Mean (SD)	28.88	(7.65)	27.56	(7.93)	27.61	(7.89)	27.81	(7.36)	29.06	(8.26)

*SD* Standard deviation, *IPLC* initial primary lung cancer, *SPLC* second primary lung cancer, *NSCLC*/*NOS* non-small cell lung cancer/not otherwise specified, *TDI* Townsend Deprivation Index (i.e., area-level socioeconomic status).

**Fig. 2:**
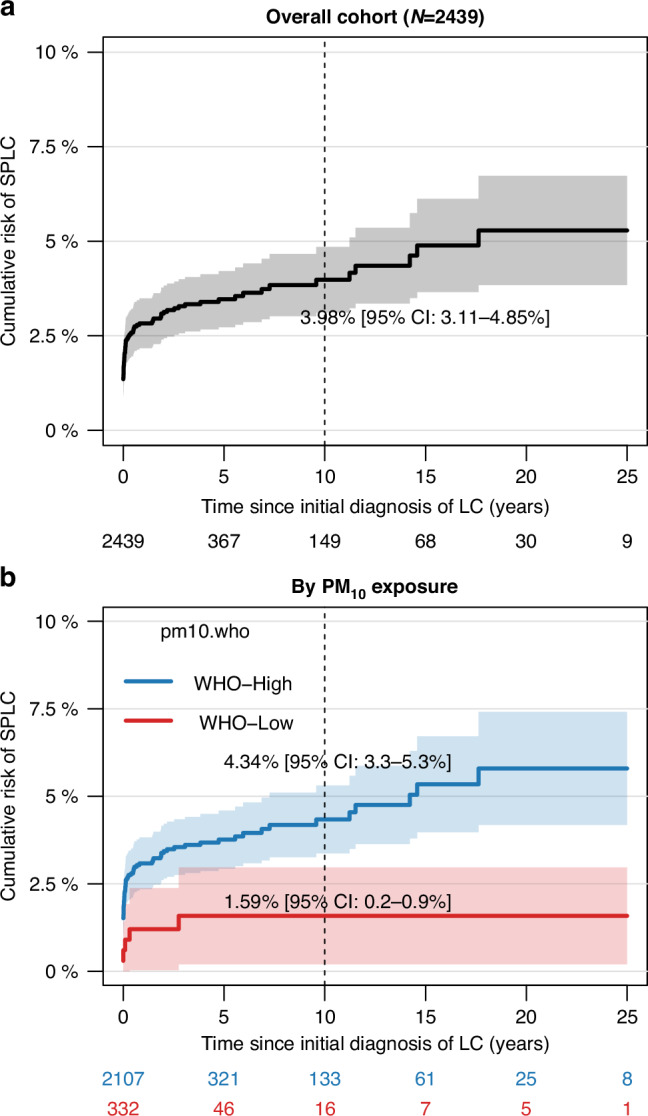
Cumulative incidence of SPLC overall and by PM_10_ exposure level. The cumulative incidence of SPLC and the 10-year cumulative incidence with 95% CI for the entire study cohort (*N* = 2439) in the upper panel (**a**), and stratified by PM_10_ levels according to the World Health Organisation’s exposure threshold—low (20 μg/m^3^) vs. high (⩾ μg/m^3^) PM_10_ exposure in the lower panel (**b**).

Of the 2439 IPLC cases in the study cohort, 92 (3.8%) developed SPLC after IPLC over 6560 person-years. The 10-year cumulative incidence of SPLC since IPLC diagnosis was 3.98% (95% confidence interval [CI]: 3.11% to 4.85%) (Fig. 2a). The 10-year cumulative incidence of SPLC among those with high PM10 (⩾20 μg/m3) was 4.34% (95% CI: 3.37% to 5.31%) vs. 1.59% (95% CI: 0.20% to 2.97%) among those with low PM10 exposure (<20 μg/m^3^) (Fig. 2b). The distribution of PM10 levels overall and among non-SPLC vs. SPLC cases is shown in Supplementary Fig. 1.

The multivariable CSC analysis indicates a clear dose-response relationship between PM10 quintiles and an increased risk of SPLC, with adjusted hazard ratios (aHR) of 1.69 (CI, 0.68–4.19), 3.49 (CI, 1.50–8.10), 4.92 (CI, 2.08–11.60), and 6.43 (CI, 2.38–17.32) for the second through fifth quintiles compared to the first quintile (Table 2). Current smoking status was also significantly associated with an increased risk of SPLC (aHR = 2.12, CI, 1.01–4.46) when compared to IPLC patients with no history of smoking (Table 2). Other air pollutants (PM2.5, PMcoarse, and PM10) measured in 2010 using different ESCAPE methods showed an increasing trend; in aHR on SPLC risk across quintiles, however, these sensitivity analyses did not achieve statistical significance (Supplemental Table 1).

**Table 2:** Association between PM_10_ and SPLC risk in the UK Biobank (*N* = 2439).

	aHR (95% CI)	*P*-value
PM_10_		
Quintile 1	(Ref)	
Quintile 2	1.69 (0.68–4.19)	0.25
Quintile 3	3.49 (1.50–8.10)	0.003
Quintile 4	4.92 (2.08–11.60)	0.0002
Quintile 5	6.43 (2.38–17.32)	0.0002
NO_2_	0.96 (0.93–0.99)	0.03
Sex		
Women	(Ref)	
Men	1.01 (0.66–1.53)	0.95
Household income^a^		
<18 K	(Ref)	
18K–31K	0.84 (0.48–1.45)	0.54
⩾31 K	0.67 (0.34–1.31)	0.24
Unknown	1.02 (0.59–1.74)	0.95
Townsend Deprivation Index^b^		
1 ( < -3.374)	(Ref)	
2 (⩾-3.374 to <-1.840)	0.81 (0.40–1.63)	0.56
3 (⩾-1.840 to <0.513)	1.46 (0.78–2.73)	0.22
4 (⩾0.513 to <3.228)	0.53 (0.24–1.13)	0.10
5 (⩾3.228)	0.69 (0.32–1.46)	0.34
Smoking Status		
Never	(Ref)	
Former	1.28 (0.61–2.67)	0.50
Current	2.12 (1.01–4.46)	0.04
Age at IPLC diagnosis	1.01 (0.97–1.04)	0.62
IPLC histology		
Small cell, Other	(Ref)	
Non-small cell lung cancer	1.35 (0.81–2.22)	0.24

^a^Individual-level socioeconomic status.

^b^Area-level socioeconomic status.

*CI* confidence interval, *aHR* adjusted hazard ratio, *IPLC* initial primary lung cancer, *NSCLC* non-small cell lung cancer, *SCLC* small cell lung cancer, *PM*_10_ particulate matter ⩽10 μm in aerodynamic diameter.

**Fig. 3:**
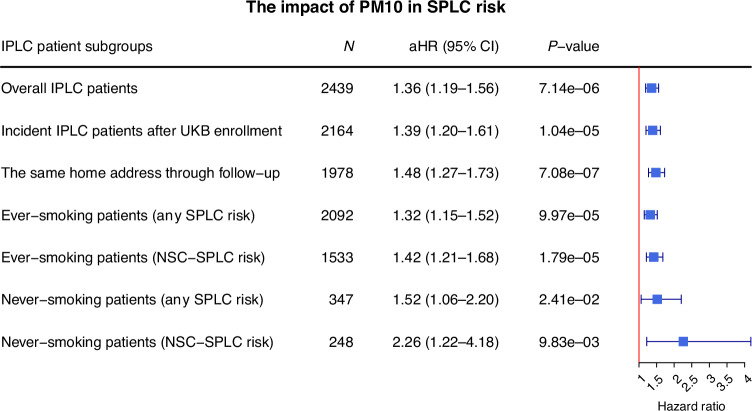
Forest plot of associations between PM_10_ and SPLC risk in overall IPLC patients and by various IPLC subgroups. Abbreviations: IPLC initial primary lung cancer, SPLC second primary lung cancer, NSC-SPLC non-small cell-second primary lung cancer.

We performed various subgroup analyses to evaluate the robustness of the main finding for the association between PM10 and SPLC risk, as well as to find the potential effect modification of PM10 impact on SPLC risk (Fig. 3). Compared to the effect in the overall cohort (aHR = 1.36, CI, 1.19–1.56; *p* = 7.1 × 10^−6^), the results solely based on incident IPLC cases (vs. the combined cohort of prevalent and incident IPLC cases in the main analysis) showed a slightly stronger association (aHR, 1.39; 95% CI: (1.20, 1.61); *p* = 1.04 × 10^−5^). To minimise potential misclassification of long-term PM10 exposure levels, we evaluated the association between PM10 and SPLC risk among only those who lived at the address recorded at baseline for at least 6 months in 2007 and who remained at this location through their IPLC diagnosis and entire follow-up period (*N* = 1,978). We observed a more pronounced association between PM10 and SPLC risk (aHR, 1.48, CI, 1.27–1.73); *p* = 7.08 × 10^−7^) (Fig. 3).

Analyses stratified by smoking status showed that although the effect of PM10 on SPLC risk was consistent across smoking status, it was more pronounced in never-smoking IPLC patients (aHR = 1.52, CI, 1.06–2.20; *p* = 0.02) compared to ever-smoking patients (aHR = 1.32, CI, 1.15–1.52; *p* = 9.9 × 10^−5^). Further, the association between PM10 and SPLC risk was particularly significant when examining non-small cell SPLC in never smokers (aHR = 2.26, CI, 1.22–4.18; *p* = 9.8 × 10^−3^) than in ever smokers (aHR = 1.42, CI, 1.21–1.68; *p* = 1.8 × 10^−5^) (Fig. 3).

## Discussion

We evaluated the association between residential air pollutant exposure and SPLC risk using the population-based and prospective UK Biobank cohort. The present study shows that residential exposure to PM10 pollution is associated with SPLC risk among lung cancer survivors. In particular, there was a clear dose-response relationship between an annual PM10 exposure and SPLC risk in lung cancer survivors in a robustly adjusted co-pollutant model using competing risk regression. This finding was consistently observed under various subgroup analyses, including a restricted analysis limited to incident IPLC cases or those staying in the same home address through follow-up. Notably, a stronger association was observed between PM10 exposure and SPLC risk in never smoking IPLC patients. Overall, our findings support PM10 as a modifiable risk factor for SPLC and lend support to incorporating PM10 in predicting SPLC risk for all lung cancer survivors, particularly those who do not smoke. Additionally, they highlight the importance of reducing PM10 levels through public health policy to further alleviate the burden of secondary malignancies in lung cancer survivors.

Particulate matter is a complex mixture of small, solid particles and liquid droplets in the air. In particular, PM10 consists of a mixture of materials that can include soot, metals, salt, and dust. Major sources of outdoor PM10 pollution include emissions from the combustion of gasoline, oil, diesel fuel, or wood, as well as dust from construction sites, landfills and agriculture, wildfires and brush/waste burning, and industrial sources [31]. Thus, coordinated efforts by regional- and national-level policymakers working in sectors like energy, transportation, waste management, urban planning, and agriculture must take place to lower PM10 levels in the atmosphere.

The impact of air pollutant exposure has been examined in association with IPLC [16, 18–22, 32, 33]. A previous study, which used data from 17 cohort studies based in nine European countries to assess the association between long-term exposure to ambient air pollution and lung cancer incidence, found a statistically significant association between the risk for lung cancer and PM10 concentration (HR = 1.22 per 10 μg/m^3^). Notably, multiple studies in the past have observed a significant association between PM2.5 (i.e., particulate matter ⩽2.5 μm in aerodynamic diameter) exposure and IPLC risk [18, 19, 22]. While the hazard ratio for the association between high PM2.5 exposure and SPLC risk in our study trended in a consistent direction, this finding was not statistically significant (Supplemental Table 1) for several reasons. First, PM10 includes both the coarse fraction (2.5–10 µm) [34] and PM2.5, and because coarse and fine particles differ in airway deposition, composition, exposure contrast, and measurement error, PM10 may capture risk signals that PM2.5 alone misses in this SPLC association analysis—even if the PM2.5 association is directionally consistent. Second, in our data, PM2.5 was only derived from the ESCAPE study using 2010 data, whereas PM10, used in the primary analysis, was obtained from the EU-wide air pollution map covering 2005–2007. Consequently, differences in pollutant source and estimation method, improvements in air quality over time, or, notably, the smaller number of SPLC cases considering temporality (i.e., censoring SPLC occurred before PM2.5 measurements) may further explain the lack of significance.

To our knowledge, this study is among the first to evaluate the dose-response relationship between air pollutant and SPLC risk among lung cancer survivors. We used data from a large, prospective cohort with high-resolution geospatial data. To accurately estimate the impact of PM10 on SPLC risk, we used a co-pollutant model with comprehensive adjustment factors including NO2, individual-level and neighbourhood-level socioeconomic factors, and tumour characteristics. The inclusion of each participant’s home location history allowed us to evaluate residential air pollution exposure with greater precision than available in other administrative datasets. We performed comprehensive subgroup analyses to evaluate the robustness of the main findings, including for the sub-population by a smoking history, among those who lived at the address recorded at baseline for at least 6 months in 2007 and who remained at this location through their IPLC diagnosis and entire follow-up period, and for only those with an incident IPLC (vs. the combined cohort of prevalent and incident IPLC).

A few limitations must be considered. The UK Biobank does not include information on IPLC stage, which has been shown to be a significant risk factor for SPLC [12]. However, for the previously ascertained SPLC risk factors (e.g., smoking status, age at IPLC diagnosis, IPLC histology) that were available in the UK Biobank, we made the relevant adjustments in each of our analyses. Another inherent limitation of the UK Biobank air pollution data is that pollutant levels were not recorded on an annual basis, even though air quality has been steadily improving worldwide. Consequently, we were unable to assess how yearly variations in air pollution levels might influence SPLC risk, leading to potential misclassification bias in exposure assessment. Therefore, future validation analyses using a more recent cohort are necessary. Also, the UK Biobank mainly includes Whites from developed countries with relatively low air pollution exposures and high levels of access to healthcare, which may limit the generalisability of the study findings to populations of different ethnicities or those who have high air pollution exposure levels. Therefore, future studies should include validating our findings in independent cohorts that consist of diverse populations. Finally, other unmeasured confounding may have influenced our results, including gene–environment interactions, occupational exposures beyond residential estimates, and indoor air pollution. These factors could lead to residual confounding or exposure misclassification. Future studies could address these limitations by incorporating personal exposure monitoring, detailed occupational and residential histories, indoor air assessments, and genetic data, as well as applying advanced causal inference methods to reduce bias.

In conclusion, using data from the large, comprehensive, and prospective UK Biobank cohort study, we observed a significant association between high PM10 exposure and the risk of SPLC among patients previously diagnosed with lung cancer. These findings suggest the importance of reducing PM10 exposure and highlight the significance of clean air action for preventing SPLC incidence.

## Supplementary information

Supplemental Material

## Data Availability

The UK Biobank data available upon request. Researchers interested in the UK biobank data may submit an enquiry online: https://www.ukbiobank.ac.uk/enable-your-research/apply-for-access.
