# Supplementary material for: Air pollution and the risk of second primary lung cancer among lung cancer survivors: the prospective UK Biobank cohort study
Source: Br J Cancer. 2026 Apr 27;135(2):283–9. doi: 10.1038/s41416-026-03454-6 (PMC13186603; doi:10.1038/s41416-026-03454-6)
Supplement: Supplementary file 1 — Supplemental Material [file 41416_2026_3454_MOESM1_ESM.docx]

# **Online Supplement**

**Title: Air Pollution and the Risk of Second Primary Lung Cancer Among Lung Cancer Survivors: The Prospective UK Biobank Cohort Study**

**Authors:** Eunji Choi, PhD,^1^* Sophia Luo, MS,^2^* Victoria Y. Ding, MS, ^2^ Anna Graber-Nadich, PhD,^2^ Julie Wu, MD, PhD,^3^ Rita Popat, PhD,^4^ Iona Cheng, PhD,^5^ Joel Neal, MD,^6^ Heather Wakelee, MD,^6^ Summer Han, PhD^2,4,7^

*: Equal Contribution

**Supplemental Method: Air pollution measures in the UK Biobank**

**Supplemental Figures**

- **Supplemental Figure 1:** Histogram of PM_10_ (2007) distribution based on low vs. high PM_10_ category. PM_10_ category is defined according to the WHO guidelines for PM_10_ in 2007: low (<20 μg/m^3^) and high (⩾20 μg/m^3^). (A) Histogram of PM_10_ (2007) distribution for the overall population (N=2785). (B) Histogram of PM_10_ (2007) distribution by non-SPLC vs. SPLC cases.

**Supplemental Tables**

- **Supplemental Table 1:** Association between Air Pollutant Variables and SPLC Risk, Adjusted for Age at IPLC Diagnosis, IPLC Histology, and Smoking Status in Multivariable Cause-Specific Cox Regression

**Supplemental Method: Air pollution measures in the UK Biobank**

Air pollution estimates for the years 2005-2007, used in the primary analyses, were obtained from EU-wide air pollution maps with a resolution of 100 m x 100 m. The x, y-coordinates of the addresses that participants provided at baseline were overlaid onto these maps (projected to British National Grid), and the corresponding air pollution concentration of the 100 m x 100 m grid cell was assigned to the coordinate. EU-wide air pollution maps were modeled based on a Land Use Regression (LUR) model for Europe, which also includes satellite-derived air pollution estimates. Further details on the model and model performance can be found in the previous work by Vienneau et al. [1].

Estimates for the year 2010 for sensitivity analyses in this study were modeled for each address using a land-use regression (LUR) model developed as part of the European Study of Cohorts for Air Pollution Effects (ESCAPE) study. ESCAPE estimates for PM in 2010 are valid up to 400 km from the monitoring area (Greater London), but the accuracy of estimates beyond this range was unknown and so these were coded as missing within the central UK Biobank dataset. For more information on the individual models, please see the ESCAPE project website: <http://www.escapeproject.eu>.

Given that these estimates from different air pollution models (EU-wide [2005-2007] vs. ESCAPE [2010]) were not designed to be combined and should not be averaged, as this may introduce bias, we selected EU-wide (2005-2007) as the primary to secure a larger number of SPLC cases in the analysis. Sensitivity analyses using ESCAPE (2010) showed similar trends but did not reach statistical significance potentially due to the lack of power from a reduced sample size.

**Reference**

[1] Vienneau D, de Hoogh K, Bechle MJ, Beelen R, van Donkelaar A, Martin RV, Millet DB, Hoek G, Marshall JD. 2013. Western European land use regression incorporating satellite- and ground-based measurements of NO2 and PM10. Environmental Science and Technology 47(23): 13555-13564.

**Supplemental Figures**

**Supplemental Figure 1:** Histogram of PM_10_ (2007) distribution based on low vs. high PM_10_ category. PM_10_ category is defined according to the WHO guidelines for PM_10_ in 2007: low (<20 μg/m^3^) and high (⩾20 μg/m^3^). (A) Histogram of PM_10_ (2007) distribution for the overall population (N=2785). (B) Histogram of PM_10_ (2007) distribution by non-SPLC vs. SPLC cases.


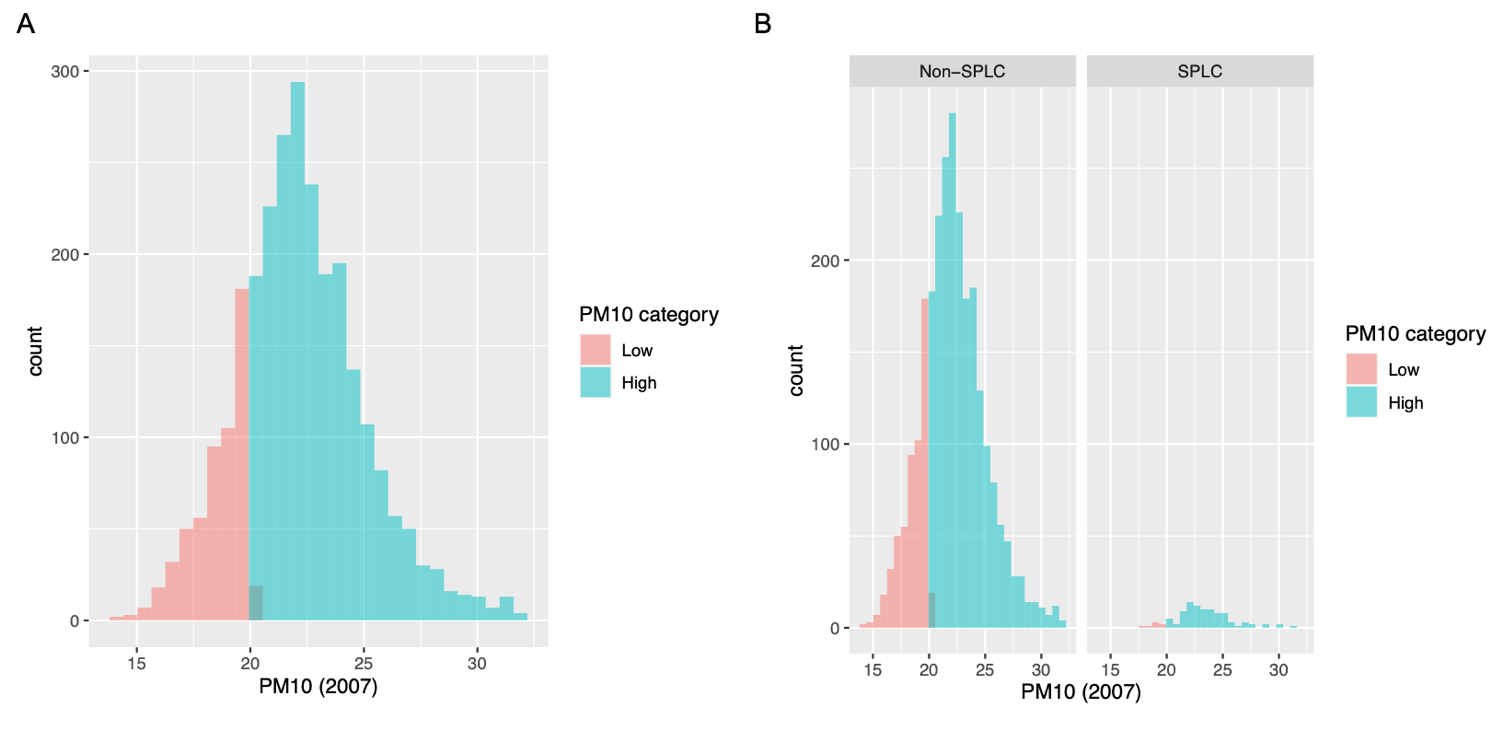


**Supplemental Tables**

**Supplemental Table 1:** Association between other air pollutant variables (i.e., PM2.5, PMcoarse, PM10 (2010) and SPLC Risk, Adjusted for Age at IPLC Diagnosis, IPLC Histology, and Smoking Status in Multivariable Cause-Specific Cox Regression

| Air Pollutant Variable | aHR (95% CI)^a^ | P-value |
| --- | --- | --- |
| PM_10_ (2010) |  |  |
| Q1 | (Ref) |  |
| Q2 | 1.33 (0.65-2.72) | 0.43 |
| Q3 | 0.95 (0.43-2.09) | 0.89 |
| Q4 | 1.41 (0.68-2.93) | 0.35 |
| Q5 | 1.77 (0.86-3.64) | 0.11 |
| PM_2.5_ (2010) |  |  |
| Q1 | (Ref) |  |
| Q2 | 0.95 (0.45-1.98) | 0.89 |
| Q3 | 0.96 (0.43-2.17) | 0.93 |
| Q4 | 1.92 (0.84-4.36) | 0.11 |
| Q5 | 1.31 (0.41-4.14) | 0.63 |
| PM_coarse_ (2010) |  |  |
| Q1 | (Ref) |  |
| Q2 | 0.63 (0.29-1.38) | 0.25 |
| Q3 | 1.33 (0.70-2.52) | 0.37 |
| Q4 | 0.78 (0.38-1.63) | 0.52 |
| Q5 | 1.40 (0.73-2.68) | 0.30 |

*Definition of abbreviations*: CI = confidence interval; aHR = adjusted hazard ratio; IQR = interquartile range; PM_2.5_ = particulate matter ⩽2.5 μm in aerodynamic diameter; PM_10_ = particulate matter ⩽10 μm in aerodynamic diameter; PM_coarse_ = coarse particulate matter between 2.5 μm and 10 μm in aerodynamic diameter; NO_2_ = nitrogen dioxide; NO_x_ = nitrogen oxides.

^a^ Model adjusted for co-pollutant (NO2), sex, household income, Townsend deprivation index, smoking status, age at IPLC diagnosis, and IPLC histology.
